# Shift work in the digital food environment: associations with nutrition knowledge and online food ordering attitudes

**DOI:** 10.3389/fnut.2026.1958118

**Published:** 2026-10-07

**Authors:** Tuğçe Özlü Karahan, Aslı Akbeniz, Gamze Çavuşoğlu, Hümeyra Kara, Ceren Ergünden, Emre Batuhan Kenger, Hatice Merve Bayram, Arda Ozturkcan

**Affiliations:** 1 Hamidiye Faculty of Health Sciences, Department of Nutrition and Dietetics, University of Health Sciences Türkiye, Istanbul, Türkiye; 2 Faculty of Health Sciences, Department of Nutrition and Dietetics, Istanbul Bilgi University, Istanbul, Türkiye; 3 Faculty of Health Sciences, Department of Nutrition and Dietetics, Fenerbahçe University, Istanbul, Türkiye; 4 Faculty of Health Sciences, Department of Nutrition and Dietetics, Istanbul Gelisim University, Istanbul, Türkiye

**Keywords:** food choice, knowledge, nutrition, online food ordering, shift work

## Abstract

**Introduction:**

Shift work may influence individuals’ online food-ordering behaviors by altering meal timing and access to food; however, the role of nutrition knowledge in this relationship remains insufficiently understood. This study aimed to examine the association between nutrition knowledge levels and attitudes toward online food ordering among adults with different work schedules. Methods: This cross-sectional study included 352 employees aged 18–64 years. Sociodemographic characteristics, work schedules, and online food-ordering behaviors were assessed using a structured questionnaire. Nutrition knowledge was evaluated using the Nutrition Knowledge Level Scale for Adults, while attitudes toward online food ordering were assessed using the Attitude Scale Toward Online Food Ordering.

**Methods:**

This cross-sectional study included 352 employees aged 18–64 years. Sociodemographic characteristics, work schedules, and online food-ordering behaviors were assessed using a structured questionnaire. Nutrition knowledge was evaluated using the Nutrition Knowledge Level Scale for Adults, while attitudes toward online food ordering were assessed using the Attitude Scale Toward Online Food Ordering.

**Results:**

Of the participants, 84.7% reported that their dietary patterns changed on working days, while 64.8% indicated that fast-food products were the most frequently ordered food category. The total attitude score toward online food ordering differed significantly according to shift type, with fixed night-shift workers having higher scores than fixed evening-shift workers (58.73 ± 11.47 vs. 50.12 ± 10.57; *p* = 0.02). The regression model explained 11.6% of the variance in attitude scores (*p* < 0.001). The food-choice score was a positive predictor (*β* = 0.270; *p* < 0.001), whereas age (*β* = −0.133; *p* = 0.010) and the basic nutrition and food–health knowledge score (*β* = −0.138; *p* = 0.010) were negative predictors. Working a fixed night shift was associated with a higher attitude score (*B* = 4.104; *p* = 0.046). The moderating effect of the food-choice score was not statistically significant (*B* = −0.213; *p* = 0.469).

**Discussion:**

The findings suggest that attitudes toward online food ordering cannot be explained solely by nutrition knowledge and that work-shift patterns, age, and food-choice tendencies should also be considered in understanding how these attitudes are shaped.

## 1 Introduction

Shift work is a widely implemented work arrangement that enables the continuous operation of the healthcare, security, transportation, industrial, and service sectors. However, working outside standard daytime hours may lead to a misalignment of the sleep–wake cycle and the timing of food intake with the endogenous circadian rhythm. Shift workers have been reported to experience disrupted meal timing, increased nighttime eating, and generally poorer diet quality (1). Similarly, a large-scale cohort study found that shift workers consumed greater amounts of processed foods, snacks, sugar-sweetened beverages, and high-fat dairy products than daytime workers (2). A systematic review similarly found that shift workers were more likely to skip meals, eat at unconventional times, and consume foods high in saturated fat and added sugars (3). Taken together, these findings suggest that the dietary consequences of shift work may be reflected more strongly in meal timing, regularity, and nutritional quality than in total energy intake alone.

Nevertheless, these dietary consequences cannot be attributed solely to circadian disruption or physiological hunger. The available literature indicates that eating behaviors among shift workers emerge from the interaction of individual and workplace-related factors, including fatigue, workload, working hours, break opportunities, coworker influence, and the availability of food in the workplace (4). Shift characteristics may further modify these influences. Rotating shifts can alter the distribution of energy intake throughout the day and disrupt established dietary patterns (5), while insufficient time for meal preparation and limited nighttime access to healthier foods may increase reliance on ready-to-eat and convenient options during both night and rotating shifts (6, 7). Consistent with this explanation, healthcare workers engaged in shift work have been reported to exhibit greater adherence to a dietary pattern characterized by high intakes of fat, meat products, and processed foods, lower adherence to healthy dietary patterns, and a lower likelihood of consuming meals regularly (8). Importantly, the magnitude and form of these dietary effects may vary according to the timing, regularity, and duration of shift schedules. Permanent night workers may experience restricted access to workplace cafeterias, supermarkets, and other conventional food outlets during working hours, whereas rotating or irregular schedules may disrupt established meal routines and make advance meal planning and food preparation more difficult (9). Similarly, long or consecutive shifts may increase time pressure and fatigue, reducing the motivation or opportunity to prepare meals before or after work. Recent evidence among shift-working healthcare professionals indicates that ultra-processed food consumption and psychological distress may differ according to shift duration, further demonstrating the relevance of working-time characteristics to food-related behavior (10). Under these conditions, the convenience, accessibility, and time-saving features of online food-ordering platforms may become particularly relevant and may be evaluated more positively by workers with irregular, rotating, or nighttime schedules. Conversely, workers with more regular and predictable schedules may have greater opportunities to plan meals, prepare food at home, and use conventional food outlets. Thus, different shift patterns may shape online food-ordering attitudes and behaviors through their effects on time availability, fatigue, meal preparation opportunities, and access to food during working hours.

The digital food environment encompasses settings in which food-related information, marketing, purchasing, and delivery are mediated through digital technologies, including online grocery retailers, restaurant websites, and food-delivery platforms (11, 12). These platforms can reduce temporal and geographical barriers to prepared food and may therefore be particularly attractive to shift workers with limited opportunities for meal preparation or access to conventional food outlets. Although their use has increased across countries and varies according to sociodemographic characteristics (13), the convenience they provide may also increase exposure to foods of low nutritional quality (12, 14, 15). Supporting this concern, an assessment of restaurant menus available through Canadian online platforms reported Healthy Eating Index scores ranging from 19.95 to 50.78 out of 100, indicating generally poor alignment with healthy eating recommendations (16). Thus, online platforms may simultaneously improve food accessibility and facilitate less healthy choices, with their ultimate influence potentially depending on users’ ability to evaluate the available options.

Nutrition knowledge encompasses an individual’s capacity to acquire and apply accurate information about foods, nutrients, healthy eating principles, and the relationship between nutrition and health. Although nutrition education can improve knowledge and promote favorable changes in the consumption of certain healthy food groups (17), food choice is not determined by knowledge alone. Available evidence indicates that nutrition knowledge, attitudes, and perceived behavioral control may strengthen intentions to select healthier foods, but these intentions do not necessarily translate into actual consumption behaviors (12). This discrepancy is also evident in digital food environments: greater motivation to eat healthily when using online food-ordering applications may coexist with higher levels of uncontrolled and emotional eating, suggesting that health-related motivation and actual eating behavior may not always be aligned (18). Collectively, these findings point to a knowledge–behavior gap in which the influence of nutrition knowledge is modified or overridden by competing factors such as taste, price, habits, accessibility, time constraints, stress, and marketing. This gap may be particularly pronounced among shift workers because occupational constraints can limit their ability to translate nutrition knowledge into healthier food choices. Indeed, despite healthcare workers’ awareness of nutrition and health, night-shift work has been associated with poorer diet quality, while heavy workloads, irregular or insufficient breaks, fatigue, and limited nighttime access to healthy foods may further encourage the selection of quick and less nutritious options (19, 20).

Nutrition knowledge may be associated with attitudes toward online food ordering because it provides a cognitive basis for evaluating the nutritional quality of foods, interpreting menu or nutrition information, and recognizing less healthy food options (21). Individuals with greater nutrition knowledge may therefore assess the foods, promotions, and information presented on online food-ordering platforms more critically and may place greater importance on the availability and visibility of healthier options (22). However, nutrition knowledge does not operate independently of the digital food environment. Convenience, price, promotional cues, product presentation, and time constraints may shape platform-related attitudes and ordering decisions, potentially weakening the translation of knowledge into behavior (11, 12). This interaction may be particularly relevant among shift workers, whose irregular schedules and limited meal opportunities can increase the relative importance of convenience. Therefore, examining nutrition knowledge together with attitudes toward online food ordering may help clarify whether nutritional understanding is reflected in individuals’ evaluations and use of these platforms.

Although previous studies have separately examined the adverse dietary consequences of shift work, the growing use of online food-ordering platforms, and the role of nutrition knowledge in food choices, these factors have rarely been investigated together among shift workers. In particular, limited evidence is available on how nutrition knowledge and perceptions of online food-ordering websites are associated with ordering behaviors in this occupational group. This represents an important gap because shift workers may rely more heavily on online food-ordering services owing to irregular schedules, limited access to healthy meals during working hours, and time constraints. Moreover, evidence from Türkiye remains scarce. By jointly evaluating nutrition knowledge, perceived website quality, and online food-ordering behaviors among shift workers, the present study provides an integrated perspective on the individual- and platform-related factors associated with food-ordering practices in this vulnerable occupational population. Nutrition knowledge may provide the cognitive basis for evaluating food options; however, the association between work schedule and attitudes toward online food ordering may also vary according to individuals’ food-choice knowledge. In particular, food-choice knowledge may influence how workers evaluate available food options under the time and accessibility constraints associated with fixed night-shift work. Therefore, the food-choice score was considered a potential moderator of the association between fixed night-shift work and attitudes toward online food ordering. Accordingly, this study aimed to compare nutrition knowledge, food-choice scores, and attitudes toward online food ordering across different shift schedules; to identify factors associated with attitudes toward online food ordering; and to examine whether the food-choice score moderated the association between fixed night-shift work and these attitudes.

## 2 Methods

### 2.1 Study design

This cross-sectional study targeted adults aged 18–64 years who were living in Türkiye and working either shift-based or fixed schedules. Data were collected between January and April 2026. Participants were recruited using a non-probability convenience sampling approach through professional communication networks and online employee groups comprising individuals working in the service, aviation, textile, security, and food sectors. An information sheet describing the study and a link to the survey were shared within these groups, and participation was voluntary. Individuals who agreed to participate were directed to an online questionnaire created using Google Forms. The required sample size was determined using an *a priori* power analysis conducted with G*Power version 3.1. The calculation was based on a comparison of two independent means, using findings from Wolska et al. (8), who reported significant differences in dietary characteristics between shift and non-shift workers. Based on the group means and standard deviations reported in that study, the estimated effect size was Cohen’s *d* = 0.352. An independent samples *t*-test with an alpha level of 0.05, statistical power of 95% indicated that a minimum of 352 participants was required. The original *a priori* sample size calculation yielded a minimum required sample of 352 participants. Because the final multivariable regression model included seven predictor parameters, the adequacy of this sample size for the regression analysis was additionally evaluated using a sensitivity analysis in G*Power. For a fixed multiple regression model with seven predictors, a total sample size of 352, *α* = 0.05, and 95% statistical power, the minimum detectable overall effect size was Cohen’s *f^2^* = 0.063. Therefore, the available sample was considered adequate for detecting small-to-moderate overall effects in the multivariable regression model.

Individuals were excluded if they were younger than 18 or older than 64 years; had a diagnosed condition requiring therapeutic dietary management, including diabetes mellitus, chronic kidney disease, celiac disease, or a diagnosed food allergy or intolerance; were following a medically prescribed restrictive diet; did not provide electronic informed consent; or submitted an incomplete questionnaire. A total of 370 individuals were reached during the data-collection period. After excluding 18 questionnaires that were incomplete, inaccurate, or otherwise unsuitable for analysis, the final analytical sample comprised 352 participants. Participation was voluntary, and electronic informed consent was obtained from all participants. The study was conducted in accordance with the Declaration of Helsinki and was approved by the Istanbul Bilgi University Human Research Ethics Committee (Decision No. 2025-10160-232; December 11, 2025).

A structured questionnaire was used to collect the study data. The first section included questions regarding the participants’ sociodemographic characteristics and working conditions. Information on age, educational level, work schedule, shift type, and duration of employment was collected. This section also included questions concerning participants’ use of online food-ordering applications.

Participants’ work schedules were determined by self-report using four predefined categories provided in the questionnaire. Participants selected the category that best represented their usual work schedule. Work schedule was self-reported and classified into four predefined categories. Fixed daytime work was defined as a standard daytime schedule generally starting in the morning (e.g., 07:00–09:00) and ending in the late afternoon (e.g., 16:00–18:00). Fixed evening shift was defined as a schedule that consistently started in the afternoon and ended around midnight (e.g., 15:00–23:00). Fixed night shift was defined as a schedule that consistently started at night and ended in the morning (e.g., 23:00–07:00). Rotating shift was defined as a work pattern in which working hours alternated between daytime, evening, and/or night shifts according to a daily, weekly, or monthly rotation schedule. Participants selected the category that best represented their usual work schedule.

Online food-ordering characteristics were assessed using self-reported categorical questions included in the structured questionnaire. Participants reported the number of online food-ordering applications they used (none, 1–3, or 4–6 applications), their frequency of online food ordering (never, 1–2 times per month, 1–2 times per week, 3–4 times per week, or every day), and the time spent using online food-ordering applications (less than 5 min, 5–15 min, or more than 15 min). Participants were also asked where they generally used these applications (at home, at work, or both), the type of food they most frequently ordered (fast food, home-style meals, diet meals, desserts/snacks, or other), and whether they checked nutritional information when placing an order (yes, no, or sometimes). Each variable was analyzed as a categorical self-reported measure.

### 2.2 Nutrition knowledge level scale for adults

The Nutrition Knowledge Level Scale for Adults was used to assess participants’ nutrition knowledge. The scale was developed by Batmaz (23), who also conducted its validity and reliability study and demonstrated that it is a valid and reliable instrument for use in the Turkish population. The scale comprises 32 Likert-type items and consists of two subscales: 20 items assessing basic nutrition and the food–health relationship and 12 items assessing food choice. Scores on the basic nutrition and food–health relationship subscale range from 0 to 80, whereas scores on the food-choice subscale range from 0 to 48. Higher scores indicate a higher level of nutrition knowledge. In the current sample, Cronbach’s alpha coefficients were 0.734 for the basic nutrition and food–health knowledge subscale and 0.813 for the food-choice subscale.

### 2.3 Attitude scale toward online food ordering

The Attitude Scale Toward Online Food Ordering was used to assess participants’ attitudes toward online food-ordering services. Developed by Yavuz and Arslan (24), the scale evaluates attitudes toward various aspects of online food-ordering systems, including the perceived quality of online food-ordering platforms, time savings, and affordability. The items are rated on a five-point Likert scale ranging from 1 (strongly disagree) to 5 (strongly agree). Higher total scores indicate a more favorable attitude toward online food ordering. In the current sample, Cronbach’s alpha coefficients were 0.815 for the perceived website quality subscale, 0.847 for the time-saving subscale, 0.940 for the affordability subscale, and 0.913 for the total scale.

### 2.4 Statistical analysis

Data were analyzed using IBM SPSS Statistics for Windows, version 30.0. Categorical variables are presented as frequencies and percentages, whereas continuous variables are reported as mean ± standard deviation. Statistical significance was set at *p* < 0.05 for all analyses. The normality of continuous variables was assessed using the Kolmogorov–Smirnov test together with skewness and kurtosis values. Given the relatively large sample size (*n* = 352) and the sensitivity of formal normality tests to minor deviations from normality in large samples, distributional characteristics were not evaluated solely on the basis of Kolmogorov–Smirnov test results. Skewness and kurtosis values between −2 and +2 were considered indicative of an acceptable approximation to a normal distribution. For samples larger than 300, absolute skewness values below 2 and absolute kurtosis values below 7 were considered indicative of no substantial departure from normality (25). In the present study, skewness values ranged from −1.613 to 0.727 and kurtosis values ranged from −1.046 to 3.345; therefore, the continuous variables were considered sufficiently close to normal for parametric analyses.

As the continuous variables included in the group comparisons satisfied the normality assumption, parametric methods were used to compare mean scores across the four independent work-schedule groups. Homogeneity of variances was assessed using Levene’s test. When the homogeneity of variance assumption was satisfied (*p* > 0.05), group differences were evaluated using conventional one-way analysis of variance (ANOVA). If the overall ANOVA was statistically significant, Bonferroni-adjusted pairwise comparisons were performed to identify which work-schedule groups differed while controlling the family-wise Type I error associated with multiple comparisons. When the homogeneity assumption was violated (*p* < 0.05), Welch’s ANOVA was used, with Games–Howell *post hoc* comparisons applied when the overall test was statistically significant. Effect sizes for group comparisons were reported using eta-squared (η^2^). Eta-squared values of approximately 0.01, 0.06, and 0.14 were interpreted as small, medium, and large effects, respectively. The chi-square test was used to examine associations between categorical online food-ordering characteristics and work schedule because both variables in these comparisons were categorical. For these analyses, participants working rotating, fixed evening, or fixed night shifts were classified as shift workers, whereas participants working fixed daytime schedules constituted the comparison group. Effect sizes for these associations were reported using Cramer’s V, with values of approximately 0.10, 0.30, and 0.50 interpreted as small, medium, and large effects, respectively.

In the regression analyses, the total attitude score toward online food ordering was the dependent variable, while the basic nutrition and food–health knowledge score and food-choice score were entered as separate continuous predictors. Univariable and multivariable linear regression analyses were performed to identify factors associated with the total attitude score toward online food ordering. Crude associations were first examined using separate univariable linear regression models. For work schedule, dummy-coded shift variables were entered simultaneously, with the fixed daytime schedule designated as the reference category. Fixed daytime work was selected as the reference category because it represented the standard non-shift work pattern and therefore provided the most appropriate baseline for evaluating differences associated with rotating, evening, and night-shift schedules. Subsequently, sex, age, basic nutrition and food–health knowledge score, food-choice score, and work schedule were entered simultaneously into a multivariable linear regression model to obtain adjusted regression coefficients. Age and sex were included as basic demographic covariates, whereas the basic nutrition and food–health knowledge score and food-choice score were included because nutrition-related knowledge and food-choice tendencies were central individual-level variables addressed by the study objectives. Work schedule was included as the principal occupational exposure of interest. All variables were entered simultaneously into the multivariable model to estimate their independent associations with the total attitude score. Regression results are presented as crude and adjusted unstandardized regression coefficients (B), 95% confidence intervals, and *p* values, with standardized regression coefficients (β) additionally reported for the adjusted model. The assumptions of multiple linear regression were assessed before interpretation of the adjusted model. Linearity and homoscedasticity were evaluated by visual inspection of the scatterplot of standardized residuals against standardized predicted values. Normality of residuals was assessed using a histogram and normal P–P plot of standardized residuals. Independence of errors was evaluated using the Durbin–Watson statistic. Potential outliers and influential observations were examined using standardized residuals, studentized deleted residuals, and Cook’s distance; standardized residuals exceeding ±3 and Cook’s distance values greater than 1 were considered potentially problematic. Multicollinearity was assessed using tolerance and variance inflation factor (VIF) values.

A moderation analysis was conducted using Model 1 of the PROCESS macro (version 5.0) to examine whether the association between fixed night-shift work and attitudes toward online food ordering varied according to food-choice score. In this model, fixed night-shift work was the independent variable, the total attitude score toward online food ordering was the dependent variable, and the food-choice score was the moderator. The fixed night-shift variable was coded as 0 = fixed daytime shift and 1 = fixed night shift, with fixed daytime work serving as the reference category. The food-choice score was mean-centered before creation of the interaction term. The interaction term (fixed night shift × food-choice score) represented the moderation effect and tested whether the difference in online food-ordering attitude scores between fixed night- and fixed daytime workers changed according to the level of food-choice score. The moderation effect was evaluated using the interaction coefficient, its 95% confidence interval, *p* value, and the additional variance explained by the interaction term (Δ*R^2^*).

## 3 Results

A total of 352 employees were included in the study, of whom 51.42% were women and 48.58% were men. Regarding educational attainment, the largest proportion of participants held a bachelor’s degree (39.49%), while 42.33% had been working in their profession for 7 years or longer. Of the participants, 45.17% worked fixed daytime shifts, 33.81% worked rotating shifts, 11.36% worked fixed night shifts, and 9.66% worked fixed evening shifts. Most participants (84.66%) reported that their dietary patterns changed on working days, indicating that work schedules were commonly accompanied by changes in usual eating routines. Although 77.27% of participants used between one and three online food-ordering applications, ordering was generally occasional: 43.47% placed orders once or twice per month and 25.85% once or twice per week. In addition, 56.53% reported spending 5–15 min on online food-ordering applications (Table 1).

**Table 1:** Distribution of participants’ sociodemographic and work-related characteristics and online food-ordering behaviors (*n* = 352).

Variable	Category	*n*	%
Sex	Male	171	48.58
	Female	181	51.42
Education Level	High school	118	33.52
	Associate degree	69	19.60
	Bachelor’s degree	139	39.49
	Postgraduate degree	26	7.39
Duration of professional experience	Less than 1 year	48	13.64
	1–3 years	83	23.58
	4–6 years	72	20.45
	7 years or more	149	42.33
Work schedule	Fixed daytime shift	159	45.17
	Rotating shift	119	33.81
	Fixed evening shift	34	9.66
	Fixed night shift	40	11.36
Change in dietary patterns on working days	Yes	298	84.66
	No	54	15.34
Number of online food-ordering applications used	None	42	11.93
	1–3 applications	272	77.27
	4–6 applications	38	10.80
Frequency of online food ordering	Never	40	11.36
	1–2 times per month	153	43.47
	1–2 times per week	91	25.85
	3–4 times per week	50	14.20
	Every day	18	5.11
Time spent using online food-ordering applications	Less than 5 min	111	31.53
	5–15 min	199	56.53
	More than 15 min	42	11.93
Most frequently ordered type of food	Fast food	228	64.77
	Home-style meals	44	12.50
	Diet meals	15	4.26
	Desserts/snacks	23	6.53
	Other	42	11.94
Checking nutritional information when placing an order	Yes	39	11.08
	No	194	55.11
	Sometimes	119	33.81

Data are presented as frequencies and percentages [*n* (%)].

Participants’ attitudes toward online food ordering and nutrition knowledge scores were compared across shift types. Perceived website quality scores did not differ significantly by shift type (*p* = 0.07). In contrast, time-saving scores differed significantly across shift types (*p* = 0.03). Bonferroni-adjusted pairwise comparisons showed that participants working fixed night shifts had higher time-saving scores than those working fixed daytime shifts (20.33 ± 4.17 vs. 17.96 ± 4.93). Affordability scores also differed significantly by shift type (*p* = 0.04), with fixed night-shift workers scoring higher than fixed evening-shift workers (24.18 ± 7.94 vs. 19.06 ± 6.98). Similarly, the total attitude score toward online food ordering varied significantly across shift types (*p* = 0.02). Participants working fixed night shifts had higher total attitude scores than those working fixed evening shifts (58.73 ± 11.47 vs. 50.12 ± 10.57). These results indicate a more favorable perception of online food ordering among fixed night-shift workers, particularly in relation to convenience and affordability. However, basic nutrition and food–health knowledge scores and food-choice scores did not differ significantly across shift types (*p* = 0.55 and *p* = 0.81, respectively), suggesting that the observed differences in attitudes were not accompanied by differences in measured nutrition knowledge (Table 2).

**Table 2:** Comparison of scores obtained from the attitude scale toward online food ordering and the nutrition knowledge level scale for adults according to shift type.

Scale/subscale (score range)	Fixed daytime shift (*n* = 159)	Rotating shift (*n* = 119)	Fixed evening shift (*n* = 34)	Fixed night shift (*n* = 40)	*p*-value	*η^2^*
Attitude scale toward online food ordering						
Perceived website quality score (4–20)	17.99 ± 2.77	18.17 ± 2.07	16.97 ± 2.46	18.28 ± 2.14	0.07	0.020
Time savings score (5–25)	17.96 ± 4.93	18.60 ± 4.80	17.41 ± 5.08	20.33 ± 4.17	**0.03** ^**α**^	0.026
Affordability score (6–30)	21.21 ± 8.22	21.31 ± 7.83	19.06 ± 6.98	24.18 ± 7.94	**0.04** ^**β**^	0.022
Total attitude score toward online food ordering score (15–75)	53.74 ± 12.60	54.53 ± 11.37	50.12 ± 10.57	58.73 ± 11.47	**0.02** ^**β**^	0.028
Nutrition knowledge level scale for adults						
Basic nutrition and food–health knowledge score (0–80)	50.45 ± 8.16	51.87 ± 9.00	51.41 ± 9.04	51.93 ± 10.79	0.55	0.006
Food choice score (0–48)	24.84 ± 5.96	25.01 ± 5.19	25.65 ± 6.40	25.90 ± 7.95	0.81	0.004

Data are presented as mean ± standard deviation. Groups were compared using one-way analysis of variance, and Bonferroni-adjusted pairwise comparisons were performed to identify significant differences. For basic nutrition and food–health knowledge and food-choice scores, for which the homogeneity assumption was violated, Welch’s ANOVA was used. *α* indicates a significant difference between the fixed daytime-shift and fixed night-shift groups; *β*: indicates a significant difference between the fixed evening-shift and fixed night-shift groups. Statistically significant *p* values are shown in bold.

Table 3 presents the comparison of online food-ordering characteristics according to work schedule. The number of online food-ordering applications used did not differ significantly between work-schedule groups (*χ^2^* = 3.55; *p* = 0.17), indicating comparable access to or adoption of these applications. In contrast, the frequency of online food ordering differed significantly according to work schedule (*χ^2^* = 18.43; *p* = 0.01). Among shift workers, 18.13% placed orders three to four times per week and 7.77% ordered every day, compared with 9.43 and 1.89%, respectively, among fixed daytime workers. Conversely, ordering once or twice per month was more common among fixed daytime workers than among shift workers (52.83% vs. 35.75%). Thus, shift workers did not use a greater number of applications, but they tended to order through them more frequently. The time spent using online food-ordering applications also differed significantly by work schedule (*χ^2^* = 10.28; *p* = 0.01). The proportion of participants who spent more than 15 min on the applications was higher among shift workers than among fixed daytime workers (15.54% vs. 7.55%), whereas spending less than 5 mins was more common among fixed daytime workers (38.99% vs. 25.39%). A significant difference was also observed between the groups in terms of where the applications were used (*χ^2^* = 10.23; *p* = 0.01). The proportion of participants who used the applications both at home and at work was 38.34% among shift workers and 22.64% among fixed daytime workers. These patterns suggest that online food-ordering applications were more integrated into the daily routines of shift workers across both settings. The most frequently ordered type of food and whether participants checked nutritional information when placing an order did not differ significantly according to work schedule (*p* = 0.80 and *p* = 0.08, respectively), indicating that the groups differed mainly in how frequently and where they used the applications rather than in the type of food ordered or their attention to nutritional information.

**Table 3:** Comparison of online food-ordering characteristics according to work schedule.

Variable	Category	Work schedule				*χ^2^*	*p*-value	Cramer’s *V*
		Shift workers (*n* = 193)		Fixed daytime workers (*n* = 159)				
		*n*	%	*n*	%			
Number of online food-ordering applications used	None	18	9.33	24	15.09	3.55	0.17	0.100
	1–3 applications	151	78.24	121	76.10			
	4–6 applications	24	12.44	14	8.81			
Frequency of online food ordering	Never	19	9.84	21	13.21	18.43	**0.01**	0.229
	1–2 times per month	69	35.75	84	52.83			
	1–2 times per week	55	28.50	36	22.64			
	3–4 times per week	35	18.13	15	9.43			
	Every day	15	7.77	3	1.89			
Time spent using online food-ordering applications	Less than 5 min	49	25.39	62	38.99	10.28	**0.01**	0.171
	5–15 min	114	59.07	85	53.46			
	More than 15 min	30	15.54	12	7.55			
Preferred location for using online food-ordering applications	At home	69	35.75	75	47.17	10.23	**0.01**	0.170
	At work	50	25.91	48	30.19			
	Both	74	38.34	36	22.64			
Most frequently ordered type of food	Fast food	122	63.21	106	66.67	1.63	0.80	0.068
	Home-style meals	27	13.99	17	10.69			
	Diet meals	9	4.66	6	3.77			
	Desserts/snacks	11	5.70	12	7.55			
	Other	24	12.44	18	11.32			
Checking nutritional information when placing an order	Yes	16	8.29	23	14.47	5.16	0.08	0.121
	No	104	53.89	90	56.60			
	Sometimes	73	37.82	46	28.93			

Data are presented as frequencies and column percentages [*n* (%)]. The chi-square test was used to compare categorical variables. Shift workers included participants working rotating, fixed evening, and fixed night shifts. Statistically significant *p* values are shown in bold.

Factors associated with attitudes toward online food ordering were examined using crude and adjusted linear regression analyses (Table 4). In the crude analyses, age was negatively associated with attitudes toward online food ordering, indicating that each one-year increase in age was associated with a 0.175-point lower attitude score (*B* = −0.175; 95% CI: −0.300 to −0.051; *p* = 0.006). Conversely, each one-point increase in the food-choice score was associated with a 0.455-point higher attitude score (*B* = 0.455; 95% CI: 0.250–0.659; *p* < 0.001). Fixed night-shift workers also had attitude scores that were approximately 5 points higher than those of fixed daytime workers (*B* = 4.989; 95% CI: 0.853–9.125; *p* = 0.018). Sex, basic nutrition and food–health knowledge, rotating-shift work, and fixed evening-shift work were not significantly associated with attitude scores in the crude analyses. After adjustment, each additional year of age was associated with a 0.160-point lower attitude score (*B* = −0.160; *β* = −0.133; *p* = 0.010), while each one-point increase in basic nutrition and food–health knowledge was associated with a 0.188-point lower score (*B* = −0.188; *β* = −0.138; *p* = 0.010). In contrast, each one-point increase in the food-choice score was associated with a 0.540-point higher attitude score (*B* = 0.540; *β* = 0.270; *p* < 0.001). Fixed night-shift workers also had attitude scores 4.104 points higher than fixed daytime workers after adjustment (*B* = 4.104; *β* = 0.109; *p* = 0.046). Sex, rotating-shift work, and fixed evening-shift work were not significantly associated with attitude scores. No evidence of problematic multicollinearity was observed, with tolerance values ranging from 0.828 to 0.968 and VIF values ranging from 1.033 to 1.208.

**Table 4:** Crude and adjusted linear regression analyses of factors associated with attitudes toward online food ordering.

Predictor	Crude *B* (95% CI)	*p*-value	Adjusted *B* (95% CI)	Adjusted *β*	*p*-value	Tolerance	VIF
Sex (Female vs. male)	−0.081 (−2.603, 2.441)	0.950	−0.780 (−3.262, 1.701)	−0.033	0.537	0.930	1.076
Age	−0.175 (−0.300, −0.051)	0.006	−0.160 (−0.281, −0.038)	−0.133	**0.010**	0.968	1.033
Basic nutrition and food–health knowledge	−0.084 (−0.226, 0.058)	0.246	−0.188 (−0.330, −0.045)	−0.138	**0.010**	0.897	1.114
Food choice	0.455 (0.250, 0.659)	<0.001	0.540 (0.330, 0.750)	0.270	**<0.001**	0.903	1.107
Rotating shift	0.794 (−2.041, 3.628)	0.582	0.473 (−2.305, 3.251)	0.019	0.738	0.828	1.208
Fixed evening shift	−3.618 (−8.036, 0.800)	0.108	−4.151 (−8.475, 0.174)	−0.102	0.060	0.876	1.141
Fixed night shift	4.989 (0.853, 9.125)	0.018	4.104 (0.075, 8.133)	0.109	**0.046**	0.874	1.144

*Dependent variable*: total attitude score toward online food ordering. Crude coefficients were obtained from univariable linear regression analyses. For work schedule, the three dummy-coded shift variables were entered simultaneously, with fixed daytime shift as the reference category. Adjusted coefficients were obtained from a multivariable linear regression model including sex, age, basic nutrition and food–health knowledge score, food-choice score, and work schedule. Male sex and fixed daytime shift were used as the reference categories. The adjusted model was statistically significant: *F* (7, 344) = 6.449; *p* < 0.001; *R^2^* = 0.116. *B*, unstandardized regression coefficient; SE, standard error; *β*, standardized regression coefficient; CI, confidence interval; VIF, variance inflation factor. Statistically significant *p* values are shown in bold.

The moderating role of the food-choice score in the association between fixed night-shift work and attitudes toward online food ordering was examined (Table 5). The overall model was statistically significant and explained 7.5% of the variance in attitude scores toward online food ordering. At the mean level of the food-choice score, participants working fixed night shifts had attitude scores that were 4.648 points higher than those of fixed daytime workers (*B* = 4.648; SE = 2.156; *t* = 2.156; *p* = 0.032; 95% CI: 0.396–8.899). However, the interaction between fixed night-shift work and the food-choice score was not statistically significant (*B* = −0.213; SE = 0.293; *t* = −0.725; *p* = 0.469; 95% CI: −0.791 to 0.365). In practical terms, although food-choice scores were directly associated with attitudes toward online food ordering, the difference between fixed night-shift and fixed daytime workers did not meaningfully change across levels of food-choice score. Thus, food-choice knowledge did not explain why fixed night-shift workers reported more favorable attitudes toward online food ordering.

**Table 5:** Results of the moderation analysis examining the role of food-choice score in the association between fixed night-shift work and attitudes toward online food ordering.

Predictor	*B*	SE	*t*	*p*-value	95% CI lower bound	95% CI upper bound
Constant	54.775	0.861	63.601	**<0.001**	53.076	56.474
Fixed night shift	4.648	2.156	2.156	**0.032**	0.396	8.899
Food choice	0.448	0.138	3.242	**0.001**	0.176	0.721
Fixed night shift × Food choice	−0.213	0.293	−0.725	0.469	−0.791	0.365

The dependent variable was the total attitude score toward online food ordering. The analysis was conducted using Model 1 of the PROCESS macro. The fixed night-shift variable was coded as 0 = fixed daytime shift and 1 = fixed night shift, with participants working fixed daytime shifts serving as the reference group. The overall model was statistically significant: *F* (3, 195) = 5.306; *p* = 0.002; *R^2^* = 0.075; adjusted *R^2^* = 0.061. The additional variance explained by the interaction term was not statistically significant: Δ*R^2^* = 0.002; Δ*F* (1, 195) = 0.526; *p* = 0.469. *B*, unstandardized regression coefficient; SE, standard error; CI, confidence interval. Statistically significant *p* values are shown in bold.

## 4 Discussion

In recent years, the widespread adoption of online food-ordering applications has increased interest in their potential influence on the food choices and dietary habits of individuals with irregular working hours. Time constraints, irregular meal schedules, and changing daily routines associated with shift work may also shape individuals’ dietary preferences (3, 4). This cross-sectional study examined the relationships among shift type, nutrition knowledge, and attitudes toward online food ordering. The main findings indicated that working fixed night shifts was associated with more favorable attitudes toward online food ordering; however, the cross-sectional design precludes any causal interpretation of this association. In the adjusted regression analysis, basic nutrition and food–health knowledge was significantly negatively associated with attitudes toward online food ordering, whereas the food-choice score was significantly positively associated with these attitudes. However, the food-choice score did not moderate the association between fixed night-shift work and attitudes toward online food ordering.

In the present study, perceived website quality scores did not differ significantly across shift types. Ease of use, reliability, payment security, clear presentation of menus, and a seamless ordering process represent fundamental features that users expect from online platforms, regardless of their working hours. Nguyen et al. (26) demonstrated that perceived ease of use, perceived usefulness, and trust in the website were important determinants of attitudes toward purchasing food online. Similarly, service quality, security, and ease of use have been reported to strengthen favorable attitudes toward online food ordering (27). In this study, individuals working fixed night shifts had higher time-saving scores than fixed daytime workers, as well as higher affordability and overall attitude scores toward online food ordering than fixed evening-shift workers. Reduced time available for grocery shopping and meal preparation, post-shift fatigue, and limited access to food during nighttime hours may influence the food choices of night-shift workers (4, 28). Furthermore, perceptions of time and cost savings have been reported to contribute to more favorable attitudes toward online food ordering (29). When online food-ordering characteristics were examined according to work schedule, shift workers were found to place orders more frequently than fixed daytime workers. They were also more likely to spend more than 15 min using online food-ordering applications and to use these applications both at home and in the workplace. These findings suggest that shift workers not only perceive online food-ordering platforms more favorably but may also integrate them more extensively into different aspects of their daily lives. In addition, basic nutrition and food–health knowledge scores and food-choice scores did not differ significantly across shift types. The literature on eating behaviors among shift workers supports this distinction between knowledge and practice. Gupta et al. (4) reported that the dietary behaviors of shift workers are shaped not only by individual knowledge and motivation but also by fatigue, sleep patterns, break opportunities, the availability of food-storage facilities, and the food options provided in the workplace. Shift workers have also been reported to skip meals more frequently, eat at unconventional times, and select foods of lower nutritional quality (3, 5).

In the present study, the adjusted regression model explained 11.6% of the variance in attitudes toward online food ordering. Age was negatively associated with attitudes toward online food ordering, with younger individuals reporting more favorable attitudes. One possible explanation for this association is that younger individuals may be more familiar with mobile applications, adopt digital services more readily, and place greater value on convenience-oriented consumption practices. However, these potential mechanisms were not directly assessed in the present study. In a study conducted among employees and students in Italy, increasing age was associated with a lower likelihood of using online food-ordering services in both groups (30). Similarly, another study involving 19,877 adults from five countries reported that individuals aged 18–29 years were approximately six times more likely to order food online than those aged 60 years or older (31). In our study, sex was not significantly associated with attitudes toward online food ordering in either the crude or adjusted analysis. Findings regarding sex differences in the existing literature appear to be inconsistent. In the five-country study, men were more likely than women to order from online restaurants, whereas sex was not a significant determinant among employees in the Italian study; among students, however, being female was associated with a higher likelihood of using online food-ordering services (30, 31). These inconsistencies should be interpreted in light of methodological and contextual differences across studies. Variations in sample characteristics and age distributions, cultural norms surrounding food purchasing, and the instruments used to assess online food-ordering attitudes and behaviors may partly explain the differing sex-related findings. In the adjusted regression model, the food-choice score was positively associated with attitudes toward online food ordering, whereas the basic nutrition and food–health knowledge score was negatively associated with these attitudes. These findings indicate associations in opposite directions but should not be interpreted as causal relationships because of the cross-sectional study design. In online ordering contexts, food price, visual appearance, delivery time, the availability of macronutrient information, and access to healthy options have been associated with consumer choices (32). Similarly, Alshahry and Alhazmi (33) identified price, food appearance, and the inclusion of a variety of vegetables in the meal as significant predictors of healthy food choice. It is noteworthy that the food-choice score positively predicted attitudes toward online food ordering, whereas basic nutrition and food–health knowledge was a negative predictor. These opposing directions may indicate that the two subscales assess distinct constructs. The negative association may reflect a more critical evaluation of the nutritional content and potential health limitations of foods offered through online platforms among individuals with greater nutrition knowledge. Indeed, menus available on online food-ordering platforms have generally been reported to have poor nutritional quality and to inadequately meet healthy eating recommendations (12, 14, 15). Thus, although individuals with greater nutrition knowledge may acknowledge the time-saving benefits and accessibility of these platforms, they may nevertheless adopt a more cautious overall attitude toward online food ordering. However, the negative association between nutrition knowledge and attitude does not necessarily indicate a lower frequency of actual online food ordering. Furthermore, although the food-choice score was a direct predictor of attitudes toward online food ordering, it did not strengthen or weaken the association between fixed night-shift work and attitude. This finding suggests that the tendency of night-shift workers to use online food-ordering services may be influenced by environmental conditions—such as time constraints, fatigue, working hours, and limited access to food at night—relatively independently of their individual food-choice tendencies.

This study has several limitations. First, owing to its cross-sectional design, causal inferences cannot be drawn regarding the relationships among the variables. In addition, the use of an online, self-reported questionnaire may have introduced recall bias. Although attitudes toward online food ordering were assessed, actual ordering records, the nutritional composition of ordered foods, and overall diet quality were not evaluated using objective methods. Furthermore, several variables that may influence attitudes toward online food ordering—including income level, weekly working hours, sleep quality, fatigue, stress, workload, break opportunities, and access to healthy foods in the workplace—were not assessed. In addition, the use of convenience sampling and voluntary participation may have introduced selection bias, as individuals who were more accessible through professional or online networks, or more interested in the study topic, may have been more likely to participate. Therefore, the sample may not be fully representative of the broader shift-working population in Türkiye, which may limit the generalizability of the findings. Future studies should incorporate objective measures, such as application usage records, actual order histories, and time-stamped digital data, to verify self-reported online food-ordering behaviors and reduce potential recall and reporting biases.

## 5 Conclusion

In this study, fixed night-shift work was associated with more favorable attitudes toward online food ordering. In the adjusted analysis, food-choice score was positively associated with these attitudes, whereas age and basic nutrition and food–health knowledge score were negatively associated. However, food-choice score did not moderate the association between fixed night-shift work and attitudes toward online food ordering. These findings demonstrate associations between attitudes toward online food ordering and the measured individual and shift-related variables; however, causal conclusions cannot be drawn because of the cross-sectional design. Work-related conditions that were not assessed in this study, including working hours, time constraints, meal-break opportunities, and access to healthy foods, warrant investigation in future research.

Future studies should examine whether these workplace conditions influence actual online food-ordering behaviors. Such evidence could inform workplace interventions involving regular meal breaks, access to healthy food options across all shifts, and improved visibility of nutritional information on online food-ordering platforms. Policymakers responsible for occupational health and labor policies could also consider regulations supporting protected meal breaks and equitable access to healthy foods across different work schedules. If inadequate workplace food access and meal-break opportunities persist, they may contribute to greater reliance on convenient and potentially less nutritious food options, with possible implications for diet quality, employee wellbeing, and occupational health inequalities. However, these potential consequences require confirmation in longitudinal studies.

## Data Availability

The raw data supporting the conclusions of this article will be made available by the authors, without undue reservation.
